# Distribution Characteristics and Pollution Assessment of Lead and Cadmium Content in Selected Dairy Farms in Jiangsu, China

**DOI:** 10.3390/vetsci12111042

**Published:** 2025-11-01

**Authors:** Yi Yang, Yan-Ni Wu, Yi-Hui Zhang, Xiang-Shun Cui, Xiao-Yang Lv, Zhi Chen, Zhang-Ping Yang, Qin-Yue Lu

**Affiliations:** 1 College of Veterinary Medicine, Yangzhou University, Yangzhou 225009, China; yangyi@yzu.edu.cn (Y.Y.); mx120220877@stu.yzu.edu.cn (Y.-N.W.); first22686@126.com (Y.-H.Z.); dx120170085@yzu.edu.cn (X.-Y.L.); zhichen@yzu.edu.cn (Z.C.); yzp@yzu.edu.cn (Z.-P.Y.); 2 Department of Animal Science, Chungbuk National University, Cheongju 28644, Republic of Korea; xscui@cbnu.ac.kr

**Keywords:** lead, cadmium, dairy farm, soil organic matter, pH value

## Abstract

**Simple Summary:**

Dairy farms are important for milk production, but they can be affected by environmental pollution. Lead and cadmium are toxic heavy metals that can harm cows and, through milk, potentially affect human health. In this study, we investigated the level of lead and cadmium pollution in three dairy farms in Jiangsu, China. We collected and analyzed soil and cow manure samples. Our results show that while lead pollution was generally low, cadmium pollution was more widespread and reached a moderate level in some areas. We also found that these metals were present in cow manure, indicating that the cows were exposed to them. Importantly, we identified that the pH level of soil is a key factor influencing the accumulation of these metals. This study provides crucial data for developing safer and more sustainable dairy farming practices to protect both animal health and milk safety.

**Abstract:**

Lead and cadmium are prevalent heavy metal toxins that contaminate the natural environment through animal husbandry and agricultural and industrial activities. Exposing dairy cows to these pollutants can have detrimental effects on milk production and quality, leading to health problems and decreasing the animals’ production performance. Therefore, investigating the distribution of lead and cadmium content and assessing the pollution levels at dairy farms are of significant theoretical and practical significance. This study determined the spatial distribution and clustering of lead and cadmium by sampling soil and feces from dairy farms in Jiangsu, China. The data obtained, in conjunction with soil data from Jiangsu Province, were used to evaluate the extent of lead and cadmium pollution at these farms. The results indicate that lead pollution levels are relatively low, whereas cadmium pollution is moderate in multiple regions. Ultimately, this study contributes to the assessment of the risks associated with lead and cadmium in dairy farming production and supports the establishment of a sustainable animal husbandry system, serving as an effective reference for subsequent ecological health farming, disease prevention, and management.

## 1. Introduction

Dairy cows are a crucial component of the dairy industry, yielding significant economic resources such as milk and dairy products. Given their substantial presence in our diets, they offer exceptional nutritional value and possess preventive and health functions for numerous diseases [1]. Therefore, the significance of the yield and quality of milk and dairy products in dairy farming cannot be overstated. These factors are subject to a range of influences, including genetic factors, feeding management levels, and environmental factors [2]. Previously, researchers concentrated mostly on enhancing genetic factors or feed nutrition levels to augment milk production and quality [3,4]. However, the escalating issue of environmental pollution has emerged as a global problem, prompting society to renew its focus on the quality and safety of milk and dairy products [5,6].

Resolving this issue is not a straightforward task. It is acknowledged that, from both a genetic and nutritional perspective, only a certain degree of prevention and treatment can be attained, and complete avoidance of quality problems caused by environmental pollution, especially heavy metals, is unfeasible. Heavy metals are a widely recognized and important form of environmental pollution, having caused serious damage to humans and the ecological environment [7,8]. Lead and cadmium are prevalent toxic heavy metal pollutants originating from agriculture, animal husbandry, and industry, and are present in the natural environment [9,10]. Exposure to toxic substances, such as lead and cadmium, can damage the mammary glands of dairy cows, thereby compromising milk production and quality, ultimately leading to health problems in the animals and decreasing production performance [11,12]. Regrettably, the efficacy of existing treatment modalities remains constrained [13]. The prevailing treatment approaches currently include chelating agent therapy and hemoperfusion therapy, with additional drug treatments also relying on chelating agent therapy [14]. In cases of chronic lead and cadmium poisoning, complete removal of toxic substances from the body is a protracted and intricate process. The therapeutic efficacy of existing treatment modalities may not be significant, especially for affected animals who have already incurred organ damage [15,16]. In addition, some chelating agents and drugs have been known to elicit side effects, such as kidney damage, gastrointestinal discomfort, and allergic reactions [17,18]. Despite ongoing efforts by researchers to develop new treatments, the results remain suboptimal. In this context, therefore, the most effective approach may be to prevent and avoid poisoning caused by lead and cadmium by limiting exposure sources and improving environmental conditions.

Of particular concern is the potential for lead or cadmium exposure at dairy farms. Though investigations into this matter have been limited, the findings have not been ideal [19,20,21,22], revealing excessive levels of these heavy metals in certain dairy farms. However, the pollution potential of these contaminants has yet to be fully assessed, precluding a comprehensive evaluation of the extent of lead or cadmium exposure. Fortunately, several extensive studies have provided valuable data for reference. For example, a study conducted in Guizhou, China, revealed the presence of cadmium exposure in the soil, with dairy farms situated in close proximity to the affected areas [23]. Another measurement conducted in the Chinese soil-crop system also showed slight lead exposure in a significant portion of agricultural land [24]. However, direct evidence regarding the exposure levels and pollution status of lead and cadmium specifically within the dairy farm environments of Jiangsu Province remains limited. Given the documented presence of these heavy metals in various environmental matrices across China, including agricultural soils, this study aims to empirically verify their levels in dairy farms within this region.

To address this gap and assess the dairy farms’ actual levels of exposure to lead and cadmium, we conducted a detailed environmental monitoring study in Jiangsu Province, China, a major agricultural and dairy production region. Previous studies have investigated heavy metals in various soils of Jiangsu, but none have focused specifically on dairy farm environments [25,26,27,28].

In recent years, several studies have identified the presence of toxic heavy metals in milk and dairy product samples. For example, a research report has highlighted the relatively high levels of lead in commercial milk from Jiangsu (with an average of 35.01 μg/kg), raw milk from Inner Mongolia (with an average of 28.15 μg/kg), and milk in Hebei (with an average of 23 μg/kg) [29]. Additionally, other investigations have revealed excessive cadmium levels in fermented milk, sterilized milk, and enzyme-modified milk from Nanchang, as well as higher cadmium levels in milk from Zhejiang [30,31]. Despite findings of previous studies indicating that the heavy metal content in milk does not exceed the established Chinese milk standards, given the ongoing escalation in milk consumption, it remains imperative to prioritize the safety of milk and dairy products.

However, the majority of studies on the presence of heavy metals have been confined to urban areas or rice paddies, with no inquiry into heavy metal content in dairy farms [32]. In view of this, this study conducted environmental sampling surveys of three dairy farms in Jiangsu. The primary objectives of this study are (1) to determine the content, distribution, and pollution levels of lead and cadmium in the soil of the selected dairy farms; (2) to determine the main factors governing the accumulation of lead and cadmium content; and (3) to evaluate the extent of pollution caused by lead and cadmium in these dairy farms.

## 2. Materials and Methods

### 2.1. Study Area

The present study was conducted in Jiangsu Province, China, a region located in the eastern part of the country. Jiangsu spans approximately 107,200 square kilometers and is predominantly characterized by plains with deep soil layers and medium-to-high fertility, making it a crucial area for agriculture and animal husbandry, which is often referred to as a “land of fish and rice”. Within this province, three dairy farms located in different cities (Yangzhou, Yancheng, and Xuzhou) were selected for research and sampling analysis. These cities were chosen because they span distinct regions of Jiangsu: Yangzhou represents the central Yangtze River Delta area, and has a developed economy and traditional agriculture; Yancheng represents the eastern coastal region, with its unique saline land ecosystems and large-scale agriculture; and Xuzhou represents the northwestern part of the province, which has a different industrial and agricultural background and is part of the North China Plain. This selection ensures that our samples cover a range of environmental and socio-economic conditions typical of dairy farming in Jiangsu, thereby enhancing the representativeness and applicability of our findings to the province.

**Figure 1:**
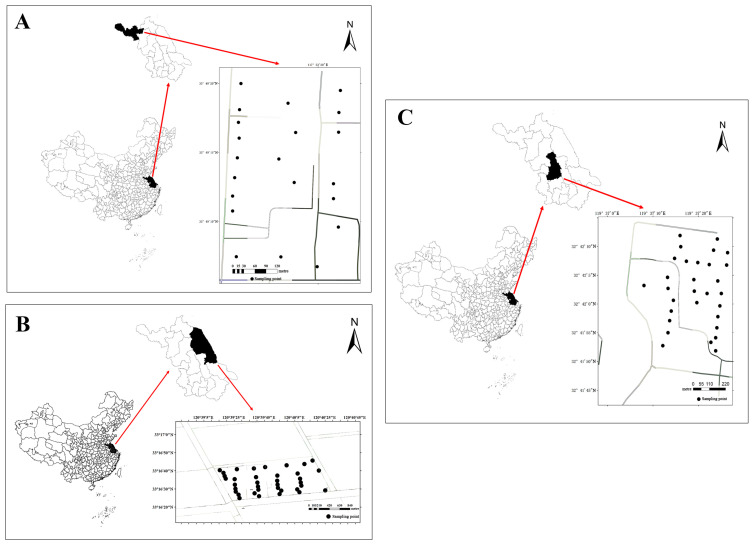
Geographic location and sampling location of dairy farms.

These farms were identified as dairy farm A (Figure 1A), dairy farm B (Figure 1B), and dairy farm C (Figure 1C).

### 2.2. Soil Sampling and Analysis

#### 2.2.1. Soil Sample Collection and Preparation

The core area for the collection of soil samples comprised miscellaneous fill, and the surrounding soil constituted the affected area. The collected samples were carefully sealed in polyethylene bags for subsequent experiments. Subsequently, they were mixed and reduced to approximately 100 g using the quartering method. After natural air drying, foreign objects, such as stones, and animal and plant residues, were removed from the soil sample. The soil samples were naturally air-dried in a clean, shaded, and well-ventilated indoor environment to prevent contamination from dust, direct sunlight (UV exposure), and precipitation. After drying, foreign objects, such as stones, and animal and plant residues, were carefully removed from each soil sample. Next, the sample was ground and pressed with a wooden rod, passed through a 2 mm nylon sieve, and thoroughly mixed. The next step was to grind the soil sample further using an agate mortar to allow it to pass through a 2 mm nylon sieve, followed by a 100-mesh nylon sieve. The resulting mixture was thoroughly combined and placed in a polyethylene bag for marking and backup. A total of 30, 36, and 25 soil samples were collected from dairy farm A, B, and C, respectively, resulting in a total of 91 samples that were available for subsequent analysis.

#### 2.2.2. Analysis of Soil pH and Organic Matter

Next, 10 g of an air-dried sample that passed through a 2 mm aperture sieve was weighed and placed in a 50 mL beaker. The soil pH measurement procedure consisted of four main stages: suspension preparation (mixing soil with water in a 2.5:1 ratio and stirring), equilibration (standing for 30 min), pH electrode measurement (reading the stabilized value in the supernatant), and routine electrode maintenance (rinsing and recalibrating every 5–6 samples).

Soil organic matter was determined using the potassium dichromate volumetric method. Briefly, a weighed soil sample (0.25–0.5 g) was digested with potassium dichromate–sulfuric acid solution at 170–180 °C for 5 min. After digestion, the remaining dichromate was titrated with a ferrous sulfate standard solution. The calculation was performed using Formulas (1) and (2):

(1) $$TOC=\frac{c\times \left(V_{0}-V\right)\times 0.003\times 1.10}{m}\times 1000$$

(2) $$OM=\frac{c\times \left(V_{0}-V\right)\times 0.003\times 1.724\times 1.10}{m}\times 1000$$

where TOC represents the mass fraction of soil organic carbon, OM represents the mass fraction of soil organic matter, *V*_0_ represents the volume of ferrous sulfate standard solution consumed for the blank test, *V* represents the volume of ferrous sulfate standard solution consumed for sample measurement, *c* represents the concentration of ferrous sulfate standard solution, m represents the mass of the sample, 0.003 is the millimole mass of 1/4 carbon atom, 1.724 is the coefficient of the organic carbon’s conversion to organic matter, and 1.10 is the oxidation correction coefficient.

#### 2.2.3. Determination of Metal Elements in Soil

An amount of 15 mL of aqua regia was transferred into a 100 mL conical flask, with the addition of 3 or 4 small glass beads. Then, the flask was placed in a glass funnel and heated on a hot plate until it reached boiling point. The inner wall of the whole conical flask was then exposed to aqua regia steam for approximately 30 min. This stage of vapor reflux was maintained to thoroughly clean and pre-condition the inner surface of the conical flask, minimizing potential contamination for the subsequent sample digestion. After the aqua regia was cooled and discarded, the inner wall was rinsed with ultrapure water and dried to be kept on standby. Next, 0.1 g of the sample was weighed (accurate to 0.0001 g) and placed into the previously prepared 100 mL conical flask for testing. Following the addition of 6 mL of aqua regia solution, the conical flask was placed into a glass funnel and heated on an electric heating plate, and the aqua regia was maintained in a state of slight boiling for 2 h. It was imperative that the aqua regia vapor refluxed on the bottle wall and glass funnel without progressing to an overly intense reaction that could result in sample overflow. After digestion, the flask stood and cooled to room temperature, followed by filtration and collection of the extract in a 50 mL volumetric flask with a slow quantitative filter paper. Following that, the glass funnel, conical flask, and filter residue were cleaned with a small amount of nitric acid solution at least three times. The washing solution was subsequently filtered and collected in a volumetric flask, which was then diluted to the appropriate scale with test water. The calculation was performed using Formula (3):

(3) $$w_{1}=\frac{\left(p-p_{0}\right)\times V\times f}{m}\times 10^{-3}$$

where *w*_1_ represents the content of metal elements in the soil sample, *p* represents the mass concentration of metal elements in the sample calculated from the standard curve, *p*_0_ represents the mass concentration of corresponding metal elements in the laboratory blank sample, *V* represents the constant volume of the sample after digestion, *f* is the dilution factor of the sample, and *m* represents the mass of the sample after sieving.

### 2.3. Fecal Sampling and Analysis

#### 2.3.1. Fecal Sample Collection and Preparation

The cow fecal samples were collected using a sterile fecal collector, followed by mixing with a sterile rod and labeling. After that, they were immediately stored at −20 °C for preservation until future analysis. Six fecal samples from six different cows were collected from each farm.

#### 2.3.2. Determination of Metal Elements in Fecal

The lead (Pb) and cadmium (Cd) content in fecal samples was determined using the same analytical procedure applied to the soil samples, namely the aqua regia digestion and subsequent elemental analysis detailed in Section 2.2.3 (Formula (3)).

### 2.4. Spatial Analysis of Lead and Cadmium in Soil

#### 2.4.1. Space Distribution

There are both regular temporal and obvious spatial characteristics in the data of heavy metal pollutants. This study employed the Kriging interpolation method via the ArcGIS (Version 10.8) platform to analyze these characteristics. The Kriging method assumed that the distance or direction between sampling points can effectively reflect the spatial correlation that can be utilized to illustrate surface changes. This method is capable of fitting mathematical functions with a specified number of points or all points within a specified radius to determine the output value of each position. In the process of data gridding, it made the interpolation results more scientific and closer to reality by considering the spatial related properties of the describing object. The calculation method is shown in Formula (4):

(4) $$\hat{Z}\left(s_{0}\right)=\sum_{i=1}^{N}\lambda_{i}Z(s_{i})$$

where *s*_0_ represents the predicted site, *N* represents the number of measured values, *Z*(*s_i_*) represents the measured value at the *i*-th position, and *λ_i_* represents the unknown weight of the measured value at the *i*-th position.

When using the Kriging method, the weight is contingent not only upon the distance between measurement points and the predicted position, but also upon the overall spatial arrangement of the measurement points. To use spatial arrangement in weights, it is necessary to quantify spatial autocorrelation. Therefore, the ordinary Kriging method employed a weight-fitting model, *λ_i_*, which depends on the spatial relationship between the measurement points, the distance from the predicted position, and the measurement values surrounding the predicted position.

#### 2.4.2. Spatial Clustering

Spatial clustering refers to the categorization of spatial data based on the similarity of attributes, wherein objects with similar attributes exhibit high similarity, while those with different attributes manifest significant differences. This study utilized analytical tools, such as “Global Moran’s I” and “Local Moran’s I”, on the ArcGIS platform to analyze spatial autocorrelation and clustering/outliers. Generally, the initial step involved computing the global Moran’s index of a given region, which provided insights into the presence of agglomeration or outliers in the space. In the presence of global autocorrelation, the local Moran’s index was a suitable tool for analyzing specific clustering or abnormal situations. The calculation method for the global Moran’s index is shown in Formula (5), and that for the local Moran’s index is shown in Formula (6):

(5) $$I=\frac{n\sum_{i=1}^{n}\sum_{j=1}^{n}w_{ij}(x_{i}-\bar{x})(x_{j}-\bar{x})}{\sum_{i=1}^{n}\sum_{j=1}^{n}w_{ij}(x_{i}-\bar{x}{)}^{2}}$$

(6) $$I_{i}=\frac{\left(x_{i}-\bar{x}\right)}{\sum_{i=1}^{n}(x_{i}-\bar{x}{)}^{2}}\sum_{j=1}^{n}w_{ij}(x_{i}-\bar{x})$$

where *n* denotes the number of sampling points in the study area, *w_ij_* denotes an element in the spatial weight matrix used to display the proximity relationship between the *i*-th and *j*-th sampling points, and *x_i_* and *x_j_* represent the lead/cadmium concentrations of the *i*-th and *j*-th sampling points, respectively.

The results obtained through the global Moran’s index range from −1.0 to +1.0, with values greater than 0 indicating spatial positive correlation and larger values indicating more significant spatial correlation. On the contrary, values less than 0 indicate spatial negative correlation, with smaller values indicating greater spatial difference. The value of 0 exhibits spatial randomness.

### 2.5. Assessment and Analysis of Lead and Cadmium Pollution in Soil

The geological accumulation index reflects the natural distribution changes in heavy metals in soil and emphasizes the historical accumulation of pollutants. Its calculation is shown in Formula (7):

(7) $$I_{geo}=\log_{2}\frac{C_{i}}{KB_{i}}$$

where *I_geo_* represents the geological cumulative index, *C_i_* represents the measured value of lead/cadmium content in the soil, *B_i_* represents the background value of lead and cadmium in the soil of Jiangsu, and *K* is used to rectify regional differences in the background values of lead and cadmium in the soil.

According to the *I_geo_* values, the degree of pollution can be classified and rated according to specific criteria. These criteria are as follows: *I_geo_* ≤ 0—no contamination; 0 < *I_geo_* ≤ 1—no contamination to moderate pollution; 1 < *I_geo_* ≤ 2—moderate pollution; 2 < *I_geo_* ≤ 3—moderate-to-severe pollution; 3 < *I_geo_* ≤ 4—severe pollution; 4 < *I_geo_* ≤ 5—severe-to-extreme pollution; *I_geo_* > 5—extreme pollution.

### 2.6. CART Decision Tree

The CART decision tree was used to output the conditional probability distribution of random variable Y, given the input random variable X. This model consists of three main components: feature selection, tree generation, and pruning. The *Gini* index was employed to identify the best feature and determine the optimal binary cutting point for that feature. Then, the feature with the smallest *Gini* coefficient and the corresponding cutting point were selected as the optimal feature and cutting point. Given a set of sample *D* with *K* categories and corresponding probabilities *P_K_*, the calculation of *Gini* index is displayed in Formula (8):

(8) $$Gini\left(p\right)=1-\sum_{k=1}^{k}P_{k}^{2}=1-\sum_{k=1}^{k}\frac{|C_{K}{|}^{2}}{\left|D\right|}$$

where *C_K_* is a subset of samples belonging to *K* in *D*, and *K* is the number of classes.

To analyze the impact of soil pH, organic matter, and lead/cadmium content in agricultural soil, the CART method was employed to construct a classification regression decision tree using soil lead/cadmium content as response variables and the aforementioned factors as predictive variables. These predictive variables were screened and analyzed to eliminate less significant influencing factors, and complex parameters were utilized for pruning.

## 3. Results and Discussion

### 3.1. Descriptive Statistics of Soil pH and Organic Matter

**Figure 2:**
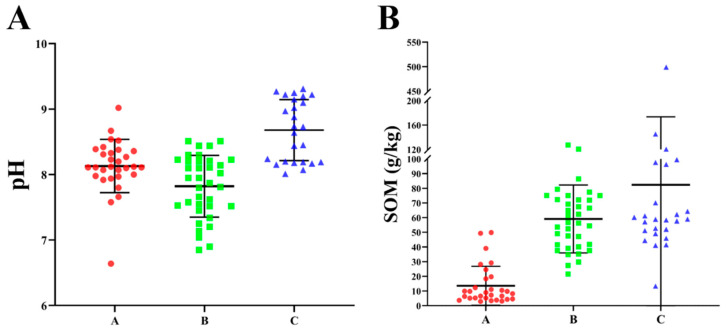
The pH value and organic matter content in soil. (**A**) The pH value in soil. (**B**) The organic matter content in soil.

The descriptive statistics of soil pH values in the study areas are shown in Figure 2A, revealing a range of 6.64 to 9.31 across three selected dairy farms. Notably, the average pH values of dairy farms A, B and C are 8.15, 7.85, 8.70, respectively, indicating an overall alkaline soil composition.

Additionally, the descriptive statistics of organic matter content in the soil of the study areas are displayed in Figure 2B, ranging from 2.90 to 499.19 g/kg across three selected dairy farms. Notably, the average values of dairy farms A, B, and C are 13.5 g/kg, 58.0 g/kg, and 82.3 g/kg, respectively, manifesting a significant difference in organic matter content among these three dairy farms. According to the second national soil survey from 2005 to 2014, the organic matter content in soil can be divided into six levels, with dairy farm A being assigned to the fourth level (10–20 g/kg) and dairy farms B and C reaching the first level (greater than 40 g/kg).

### 3.2. Descriptive Statistics of Lead and Cadmium Concentrations in Soil

**Figure 3:**
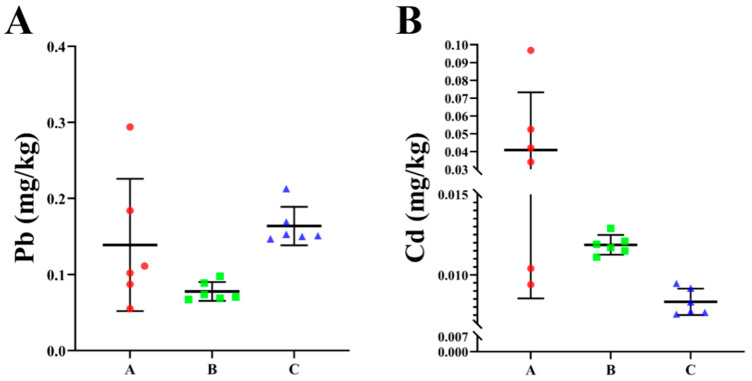
Lead (**A**) and cadmium (**B**) content at dairy farms.

The original data, following logarithmic transformation, exhibit conformity to a normal distribution. As shown in Table 1, dairy farm A displays lead content ranging from 15.0 to 29.0 mg/kg, with an average value of 18.8 mg/kg, and cadmium content ranking from 0.04 to 0.40 mg/kg, with an average value of 0.10 mg/kg. Table 2 presents the range of lead content for dairy farm B, which varies from 17.4 to 28.8 mg/kg, with an average value of 21.2 mg/kg. Additionally, the cadmium content in this farm ranges from 0.07 to 0.15 mg/kg, with an average value of 0.11 mg/kg. As shown in Table 3, the lead content of dairy farm C ranges from 13.5 to 23.0 mg/kg, with an average value of 16.3 mg/kg, and the cadmium content clusters between 0.09 and 0.247 mg/kg, with an average value of 0.14 mg/kg. These data reveal uneven lead distribution among these three dairy farms, with dairy farm B exhibiting the highest distribution and dairy farm C exhibiting the lowest distribution. By contrast, the cadmium distribution appears to be relatively average. To investigate the impact heavy metals’ presence at dairy farms may have on dairy cows, fecal samples from three dairy farms were collected and tested. The results are shown in Figure 3. Despite the lower lead and cadmium content in cow feces compared to soil, all samples tested positive for the presence of these two substances, indicating that the lead and cadmium in the environment may have entered the cows’ bodies.

### 3.3. Spatial Distribution of Lead and Cadmium Concentrations in Soil

**Figure 4:**
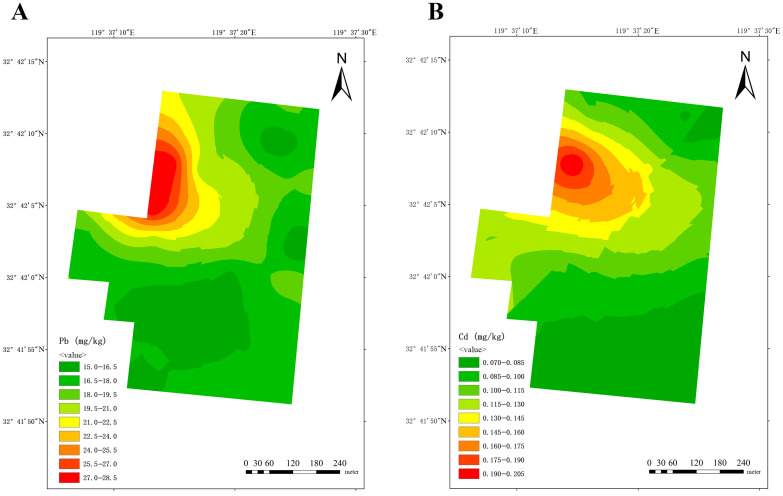
Spatial distribution of lead (**A**) and cadmium (**B**) at dairy farm A.

Utilizing data pertaining to lead and cadmium content obtained from soil from three dairy farms, a spatial distribution map of lead and cadmium content was constructed using the ordinary Kriging interpolation method. Figure 4 displays the results for dairy farm A, exhibiting a discernible regional pattern of decreasing lead and cadmium concentrations from the northwest to the southeast.

**Figure 5:**
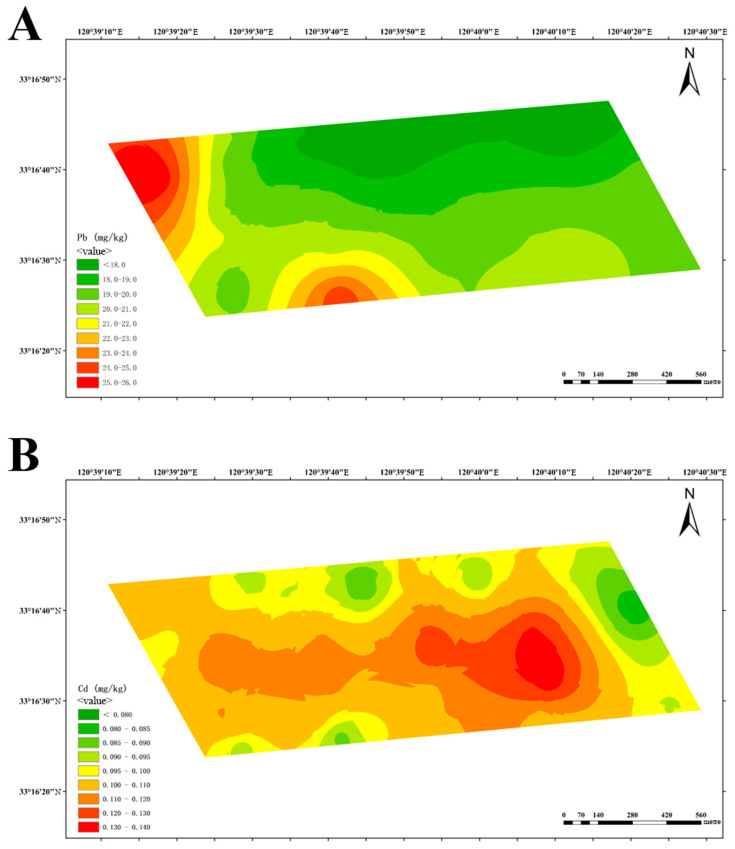
Spatial distribution of lead (**A**) and cadmium (**B**) at dairy farm B.

At-dairy farm B, elevated lead levels were detected in the northwest and south, areas which are adjacent to drainage ditches (Figure 5A). The distribution of cadmium exhibited a central diffusion pattern, with the highest concentration in the cow playground (Figure 5B).

**Figure 6:**
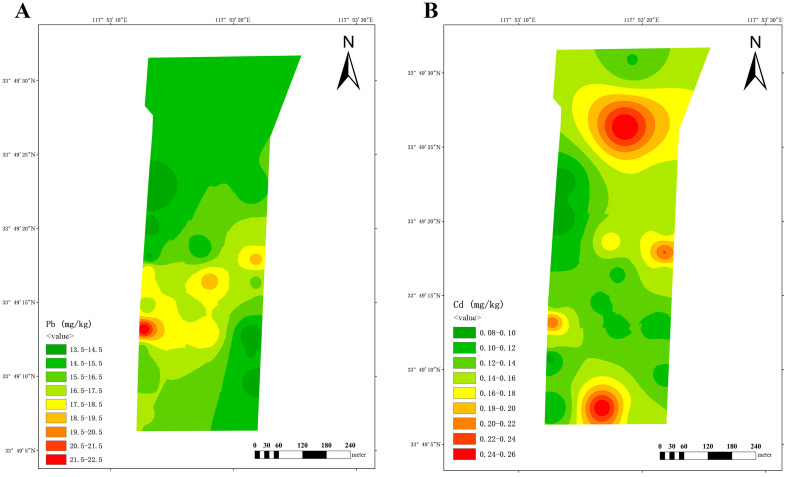
Spatial distribution of lead (**A**) and cadmium (**B**) at dairy farm C.

At dairy farm C, a single region in the southwest exhibited the highest lead content. Cadmium levels were elevated in the southern (farm entrance) and northern (near biogas digesters) regions (Figure 6).

The aforementioned findings indicate that the high distribution of lead and cadmium content in dairy farms is predominantly attributed to historical residues and geographical location.

### 3.4. Spatial Clustering of Lead and Cadmium Concentrations in Soil

**Figure 7:**
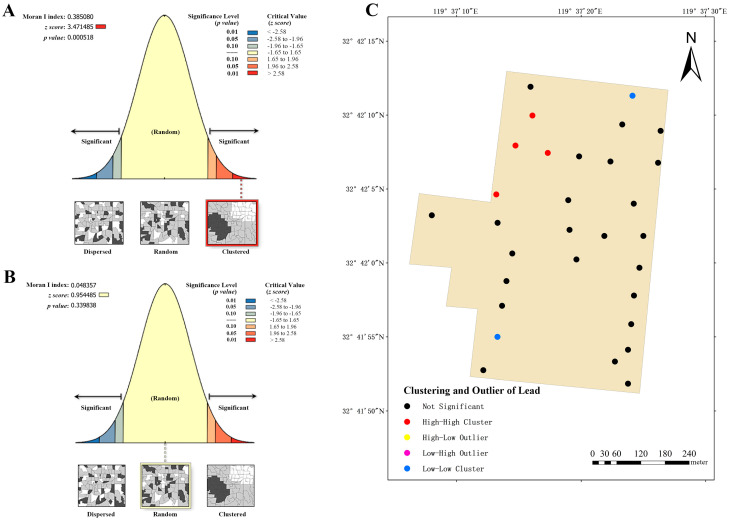
Analysis of global and local Moran’s I index for dairy farm A. (**A**) Global Moran’s I index analysis of lead. (**B**) Global Moran’s I index analysis of cadmium. (**C**) Local Moran’s I index analysis of lead.

Based on the content and related attributes of lead and cadmium in the soil of three dairy farms, the global Moran’s index was employed to evaluate the spatial distribution expressed patterns, whether they were clustered, discrete, or random. The global Moran’s index of lead distribution for dairy farm A shows a positive value of 0.385080 (Figure 7A), signifying a clustering trend. Combining the *z*-score (3.471486, >2.58) and *p*-value (0.000518), it is evident that the spatial patterns observed at a 99% confidence level are unlikely to be generated randomly, and there is a highly significant spatial autocorrelation in the lead content of the soil. Consequently, this index was used to analyze the examination of clustering and outliers (Figure 7C) in the aforementioned area, revealing the presence of four high–high clusters and two low–low clusters. This observation indicates that high–low clustering is more probable in these areas. Similarly, the global Moran’s index was employed to evaluate the cadmium distribution at dairy farm A (Figure 7B), yielding an index value of 0.048357 (>0). However, its *z*-score (0.954485, between −1.65 and 1.65) and *p*-value (0.339838) did not indicate significant spatial autocorrelation.

**Figure 8:**
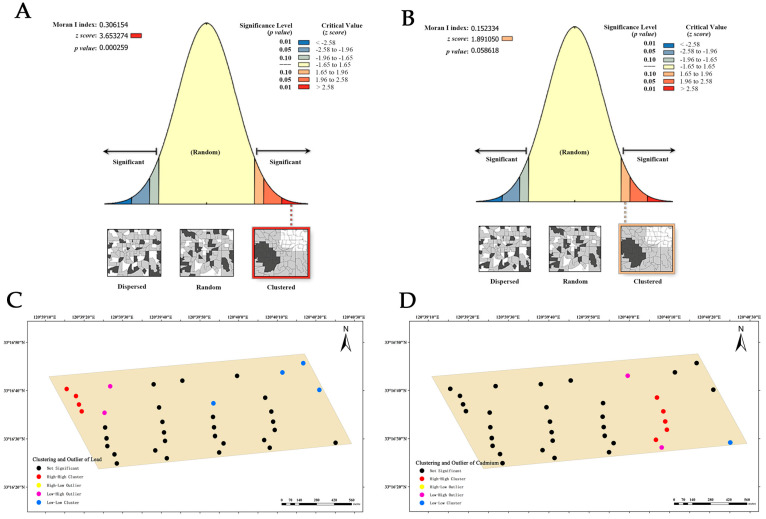
Analysis of global and local Moran’s I index at dairy farm B. (**A**) Global Moran’s I index analysis of lead. (**B**) Global Moran’s I index analysis of cadmium. (**C**) Local Moran’s I index analysis of lead. (**D**) Local Moran’s I index analysis of cadmium.

Dairy farm B exhibits a clustering trend in the global Moran’s index of lead distribution, with a value of 0.306154 (Figure 8A). As evidenced by the combination of the *z*-score (3.653274, >2.58) and *p*-value (0.000259), it is conspicuous that the spatial patterns observed at 99% confidence level are unlikely to be randomly generated. This suggests a highly significant spatial autocorrelation in the lead content of the soil. Therefore, the local Moran’s index is used to analyze its clustering and outlier (Figure 8C), revealing the presence of four high–high clustering points, two low–high clustering points, and four low–low clustering points. This observation indicates a higher likelihood of high–low clustering occurrence in these areas. The analysis of cadmium distribution at this dairy farm showed a global Moran’s index of 0.152334 (Figure 8B), *z*-score of 1.891050 (ranging from 1.65 to 1.96), and *p*-value of 0.058618 (ranging from 0.05 to 0.10). These results indicate that the spatial clustering trend observed at a 90% confidence level is unlikely to be randomly generated, and there is significant spatial autocorrelation in the cadmium content in the soil. Furthermore, the local Moran’s index analysis (Figure 8D) identified five high–high clustering points, two low–high clustering points, and one low–low clustering point in the dairy farm, indicating a greater likelihood of high–low clustering occurrence in these areas.

**Figure 9:**
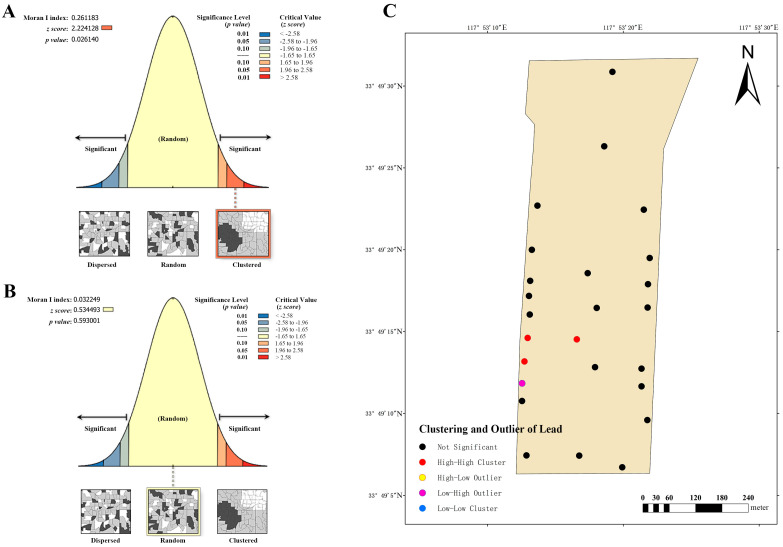
Analysis of global and local Moran’s I index at dairy farm C. (**A**) Global Moran’s I index analysis of lead. (**B**) Global Moran’s I index analysis of cadmium. (**C**) Local Moran’s I index analysis of lead.

Dairy farm C also exhibits a clustering trend in the global Moran’s index of lead distribution, at 0.261183 (Figure 9A). Combining the *z*-score (2.24128, ranging from 1.96 to 2.58) and *p*-value (0.026140), it is apparent that spatial patterns observed at a 95% confidence level are unlikely to be randomly generated. This suggests a significant spatial autocorrelation in the lead content of the soil. Therefore, the local Moran’s index was employed to further analyze its clustering and outlier (Figure 9C), revealing the presence of three high–high clustering points and one low–high clustering point. These findings indicate that high–low clustering is more likely to occur in these areas. The cadmium distribution of this farm was evaluated by the global Moran’s index (Figure 9B), and the findings were similar to those for dairy farm A. Despite its Moran’s index value of 0.032249 (>0), its *z*-score (0.534493, between −1.65 and 1.65) and *p*-value (0.593001) did not indicate noteworthy spatial autocorrelation.

Taken together, these results indicate that the lead distribution for all three dairy farms exhibits a clustering trend, with a high level of confidence indicating that the trend is not randomly generated and possesses significant spatial autocorrelation. Conversely, the distribution of cadmium at dairy farms A and C is either absent or exhibits a very low spatial autocorrelation, which may be influenced by other factors.

### 3.5. Evaluation of Lead and Cadmium Pollution in Soil

**Figure 10:**
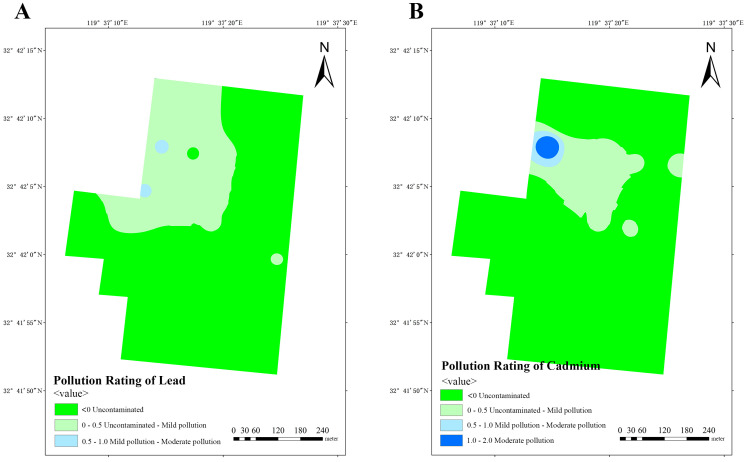
Pollution ratings of lead (**A**) and cadmium (**B**) at dairy farm A.

**Figure 11:**
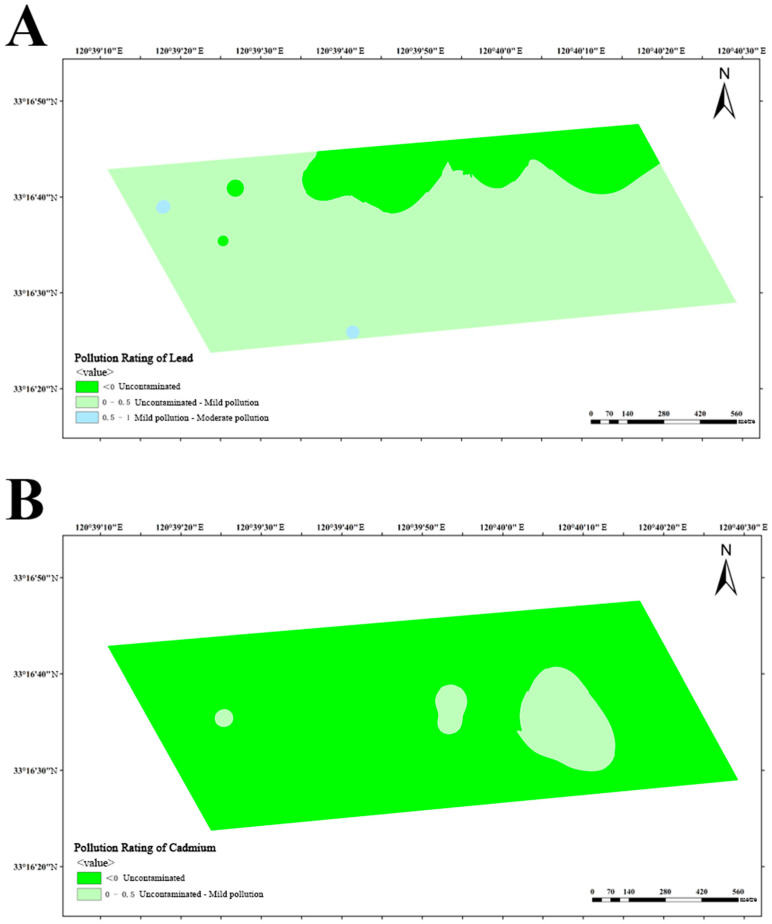
Pollution ratings of lead (**A**) and cadmium (**B**) at dairy farm B.

**Figure 12:**
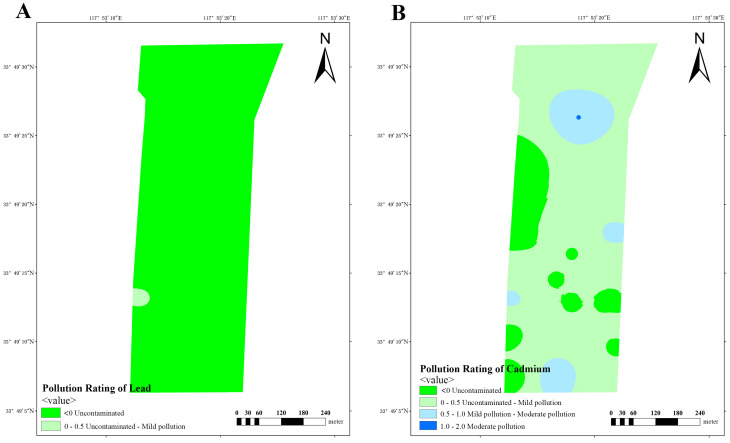
Pollution ratings of lead (**A**) and cadmium (**B**) at dairy farm C.

Based on the obtained data, calculation of *I_geo_* was performed in three dairy farms, subsequently enabling the classification of soil pollution levels in these farms. The analysis of lead pollution reveals that dairy farm A exhibits a limited number of areas with mild-to-moderate pollution, while the majority of areas remain uncontaminated or lightly polluted (Figure 10A). Similarly, dairy farm B also manifests similar results, with a small number of areas exhibiting mild-to-moderate pollution (Figure 11A). Conversely, dairy farm C demonstrates a relatively low lead distribution, with most of its areas being uncontaminated and only a small portion being lightly polluted (Figure 12A).

The impact of cadmium pollution is not as optimistic as that of lead. In dairy farm A, certain areas exhibit a level of pollution that ranges between mild and moderate (Figure 10B). A portion of dairy farm B displays a contamination level between uncontaminated and lightly polluted (Figure 11B). A small portion of dairy farm C displays a moderate level of pollution, with multiple areas experiencing mild-to-moderate levels of pollution. Most areas within this farm vary from uncontaminated to lightly polluted, with few areas remaining completely uncontaminated (Figure 12B).

The aforementioned findings indicate that three dairy farms exhibit varying levels of lead or cadmium pollution. Dairy farming is a crucial agricultural industry that offers a diverse array of dairy and meat products. However, the acceleration of urbanization and industrialization has resulted in environmental contamination in numerous regions.

### 3.6. The Main Controlling Factors for Lead and Cadmium Accumulation in Soil

**Figure 13:**
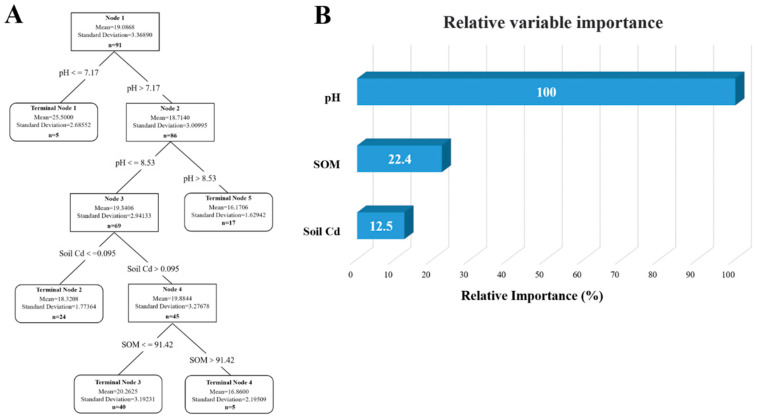
Analysis of influencing factors of lead accumulation in soil of dairy farm. (**A**) Decision tree analysis of factors affecting lead content in dairy farm soils. (**B**) The relative importance of the various influencing factors.

**Figure 14:**
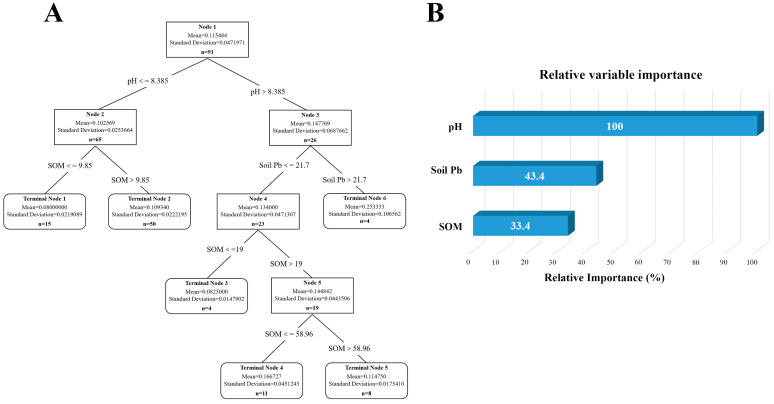
Analysis of influencing factors of cadmium accumulation in soil of dairy farm. (**A**) Decision tree analysis of factors affecting cadmium content in dairy farm soils. (**B**) The relative importance of the various influencing factors.

A model was constructed for analysis based on previously obtained soil data. The results are shown in Figure 13, with Figure 13A depicting the mutual influence of different factors on soil lead content. By calculating the importance of relative variables, it is determined that the content and distribution of lead are mainly influenced by soil pH and organic matter content, with pH accounting for 100% of the effect (Figure 13B). The analysis of cadmium is illustrated in Figure 14A, revealing that soil pH, soil lead content, and organic matter content are primary determinants of content and distribution of cadmium, with pH accounting for 100% of the influence (Figure 14B). Many studies have pointed out an interactive relationship between lead and cadmium. However, model analysis has revealed that a statistically significant correlation between lead content in soil and cadmium is absent, whereas a notable correlation exists between cadmium content in soil and lead. In addition, the pH value was identified as the most significant factor affecting the content of both lead and cadmium in the soil.

## 4. Discussion

The concurrent presence of lead and cadmium in the farm environment (soil) and in cow feces found in our study suggests that the heavy metal content in milk could potentially be influenced by environmental exposure. In this study, we assessed the lead and cadmium content of soil from three dairy farms in Jiangsu Province and identified the presence of lead and cadmium in the environment, with certain areas exhibiting moderate pollution levels. A noteworthy aspect of this study is that it replicated the aforementioned detection in cow feces, revealing the presence of lead and cadmium in samples, which is consistent with previous findings. These findings suggest that a proportion of the lead and cadmium in the environment has entered the cows’ bodies. Thus, monitoring the environmental pollution caused by toxic metals in dairy farms is of paramount significance.

As one of Earth’s most vital components, soil has numerous functions that are necessary for ecosystems and for humans [33]. It plays a core role in supporting biodiversity, facilitating agricultural production, promoting plant growth, and enabling animal and human habitation. Additionally, soil serves as a critical mechanism for mitigating climate change by sequestering carbon and nitrogen, while also protecting environmental quality by regulating the disposal, fate, and decontamination of toxic substances [34]. Beyond its role as a source of nutrients and carbon for plant growth and microbial function, soil also serves as valuable a tool for removing pollutants from agricultural, industrial, and mining activities [35]. Additionally, its composition and physicochemical properties, including its mineral and organic matter content, pH value, and surface area, are pivotal factors that influence the movement, retention, and remediation of pollutants. These factors ultimately determine the fate of pollutants in the soil [36,37]. In recent times, numerous studies have committed to identifying indicators that can facilitate the rapid detection of heavy metal contamination in soil [38,39]. This study focused on the detection and analysis of soil organic matter concentration and pH value. The results show a strong correlation between the pH value and the presence of lead and cadmium in the soil, as well as a significant correlation with soil organic matter concentration. These results suggest that pH can serve as a potential predictive indicator for soil heavy metal pollution resulting from lead and cadmium.

The spatial distribution patterns revealed in this study provide valuable insights into the potential sources of heavy metal pollution around dairy farms. In Farm A, the clear northwest-to-southeast gradient (Figure 4) coincides with historical land use records indicating that the northwestern area was previously farmland with known lead and cadmium contamination, suggesting historical residue as a primary source. In Farm B, the elevated lead levels adjacent to ditches (Figure 5A) strongly indicate transport through water flow, while the central diffusion pattern of cadmium around the cow playground (Figure 5B) points to pollution during the feeding process, possibly from feed additives or manure accumulation. For Farm C, the co-location of the highest quantities of lead and cadmium in the southwest (Figure 6) aligns with historical factory sites, implicating industrial legacy as a key contributor. Additionally, the high prevalence of cadmium around the farm’s entrance (likely from road dust) and near biogas digesters (potentially from biogas slurry accumulation) further illustrate the complexity of contamination pathways in agricultural environments.

Several studies have evidenced a significant positive correlation between the content of lead and cadmium in soil. For example, Lai et al. observed a significant positive correlation between the content of lead and cadmium in soil from Jiangxi [40]. Similarly, Naeem et al. reported comparable findings in urban farmland in Pakistan [41]. These studies indicate that the co-occurrence of lead and cadmium in soil can potentially affect their morphological transformation, migration and transformation, bioavailability, and other related factors. Specifically, lead exerts a certain impact on the adsorption and migration of soil, with its predominant forms being oxidized and reduced. The oxidized state exhibits lower biological availability and is less easily assimilated by plants, while the reduced state displays higher biological availability and is more readily absorbed by plants. Nonetheless, other elements in the soil, such as cadmium, can modulate the form and bioavailability of lead. For example, Bagheri et al. discovered that the presence of cadmium in soil can result in a reduced form being present in lead, thereby increasing plants’ uptake of lead [42]. On the other hand, cadmium can affect soil microbial activity and plant growth, thereby affecting the form and bioavailability of lead in the soil. Xu et al. observed a negative correlation between the cadmium content in soil and soil microbial activity, indicating that cadmium inhibits the growth and metabolism of soil microorganisms, thereby affecting their conversion and adsorption of lead in soil [43]. This study examined the interaction between soil lead and cadmium content in three dairy farms, revealing that cadmium content was significantly associated with the presence of lead, while lead content did not appear to have a significant relationship with the presence of cadmium. However, the specific mechanism necessitates further exploration.

This study has some limitations. Our evaluation focused on three selected dairy farms, which, while representative of different areas within Jiangsu Province, means that the findings should be extrapolated to a broader regional or national scale with caution. Nevertheless, the results provide a crucial foundation and a reliable methodological framework for assessing heavy metal pollution in dairy farms, which can be compared with future studies in other regions. This study focused primarily on soil and fecal matrices as direct indicators of environmental exposure and potential bovine uptake of lead and cadmium. This approach was chosen because manure provides a more direct and aggregate measure of the total dietary intake of pollutants, including those from contaminated feed, water, and soil. It serves as a sensitive early-warning biomarker, signaling exposure before metals accumulate to detectable or hazardous levels in milk. The transfer of heavy metals from feed to milk involves complex metabolic processes and barriers within the mammary gland, which can significantly reduce and vary their secretion [44,45]. Therefore, analyzing manure offers a more stable and comprehensive reflection of the herd’s overall exposure to environmental lead and cadmium. While water sources represent a critical exposure pathway and were beyond the scope of this initial investigation, we fully acknowledge their significant implications for animal and human health. Future research should indeed incorporate comprehensive water quality monitoring (including drinking water for cows and wastewater runoff) to construct a more holistic environmental risk assessment model for dairy farms.

## 5. Conclusions

**Figure 15:**
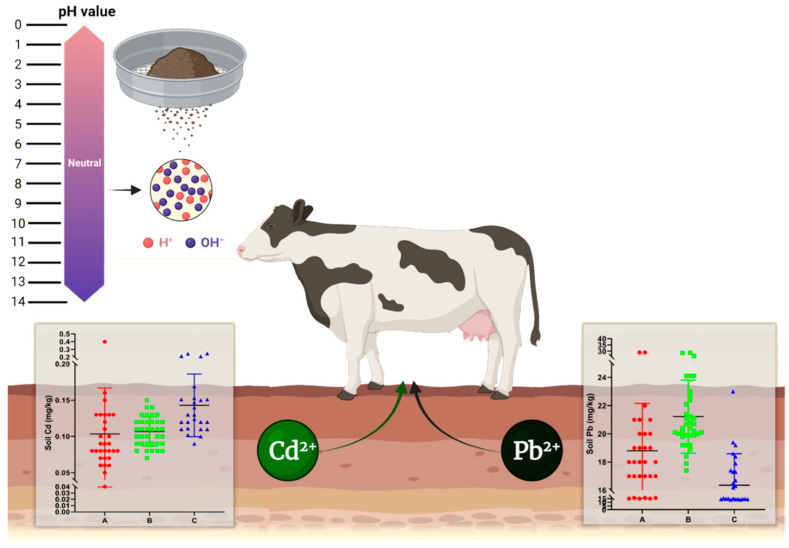
The distribution characteristics and pollution assessment of lead and cadmium content in selected dairy farms in Jiangsu, China.

This study conducted soil and fecal sampling and analysis of three dairy farms located in Jiangsu Province. The results indicate that there is a certain amount of lead and cadmium distribution in all three dairy farms, as well as in feces collected from these farms. High distributions of lead and cadmium content were also observed, with some exhibiting a clustering trend with spatial autocorrelation. Then, based on obtained soil data, a pollution assessment was conducted on the lead and cadmium content of these three farms. The findings reveal that some areas have been moderately polluted with lead/cadmium, while most areas have been either uncontaminated or range between uncontaminated and lightly polluted. This highlights the need to address the occurrence of cadmium exposure during the breeding process. Additionally, CART decision tree analysis shows that soil pH and organic matter significantly influence the lead and cadmium content. More importantly, the successful application of the CART model in this study highlights its value as a practical and interpretable tool for identifying the key controlling factors of soil heavy metal pollution. This approach can provide dairy farmers and environmental managers with clear insights for targeted monitoring and precision intervention, thereby promoting healthier and more sustainable farming practices. A comprehensive overview of the distribution characteristics and pollution assessment of lead and cadmium at the studied dairy farms is summarized in Figure 15.

## Figures and Tables

**Table 1:** Lead and cadmium content in soil from dairy farm A.

Id	Lead (mg/kg)	Cadmium (mg/kg)
1	21.000	0.040
2	18.000	0.050
3	22.000	0.070
4	15.000	0.060
5	17.000	0.060
6	29.000	0.400
7	19.000	0.130
8	21.000	0.160
9	19.000	0.130
10	19.000	0.150
11	29.000	0.100
12	21.000	0.130
13	17.000	0.110
14	18.000	0.100
15	20.000	0.120
16	20.000	0.130
17	19.000	0.130
18	15.000	0.090
19	18.000	0.090
20	16.000	0.090
21	20.000	0.080
22	16.000	0.080
23	17.000	0.080
24	15.000	0.080
25	18.000	0.070
26	17.000	0.080
27	17.000	0.070
28	16.000	0.080
29	17.000	0.080
30	18.000	0.060

**Table 2:** Lead and cadmium content in soil from dairy farm B.

Id	Lead (mg/kg)	Cadmium (mg/kg)
1	26.10	0.11
2	19.20	0.09
3	18.90	0.09
4	17.40	0.08
5	19.20	0.08
6	17.90	0.09
7	18.40	0.09
8	28.80	0.12
9	24.10	0.08
10	24.10	0.10
11	19.20	0.13
12	21.40	0.12
13	19.90	0.13
14	20.10	0.14
15	20.70	0.07
16	24.10	0.11
17	20.80	0.11
18	19.90	0.13
19	21.10	0.13
20	20.40	0.10
21	20.10	0.12
22	21.10	0.11
23	19.80	0.14
24	20.70	0.12
25	22.10	0.10
26	20.00	0.10
27	22.40	0.15
28	20.00	0.11
29	23.00	0.11
30	21.20	0.12
31	20.10	0.12
32	19.90	0.08
33	28.50	0.08
34	20.20	0.10
35	22.70	0.09
36	20.40	0.09

**Table 3:** Lead and cadmium content in soil from dairy farm C.

Id	Lead (mg/kg)	Cadmium (mg/kg)
1	14.900	0.124
2	15.100	0.247
3	13.500	0.100
4	15.200	0.151
5	14.200	0.090
6	17.300	0.153
7	14.300	0.110
8	19.200	0.213
9	18.400	0.110
10	16.500	0.150
11	17.900	0.140
12	17.300	0.130
13	17.400	0.120
14	19.400	0.120
15	23.000	0.210
16	18.600	0.120
17	13.800	0.110
18	16.200	0.151
19	14.500	0.130
20	15.700	0.102
21	16.700	0.111
22	16.300	0.244
23	14.100	0.120
24	14.600	0.147
25	14.900	0.169

## Data Availability

The original contributions presented in this study are included in the article. Further inquiries can be directed to the corresponding author.
